# Hypopituitarism and cranial nerve involvement mimicking Tolosa-Hunt syndrome as the initially presenting feature of diffuse large B-cell lymphoma: a case report

**DOI:** 10.1186/s12902-022-00973-0

**Published:** 2022-03-14

**Authors:** Shohei Kishimoto, Shuhei Morita, Chiaki Kurimoto, Chie Kitahara, Tomoya Tsuji, Shinsuke Uraki, Ken Takeshima, Yasushi Furukawa, Hiroshi Iwakura, Hiroto Furuta, Masahiro Nishi, Taka-aki Matsuoka

**Affiliations:** The First Department of Medicine, Wakayama Medical University, Wakayama, Japan

**Keywords:** Hypopituitarism, B-cell lymphoma, Tolosa-Hunt syndrome

## Abstract

**Background:**

Early diagnosis of lymphoma involving the central nervous system is sometimes difficult but emergent to avoid the delay of therapeutic initiation. Pituitary insufficiencies are usually associated with lymphoma in the pituitary gland. There have been no cases of lymphoma originating from extra pituitary gland with hypopituitarism that simultaneously presenting unilateral upper cranial nerve palsies and ophthalmalgia. These symptoms are mostly caused by neoplastic involvement of the skull base or benign diseases such as Tolosa-Hunt syndrome (THS). We report a case of lymphoma with unique clinical courses initially presenting hypopituitarism and symptoms mimicking THS with a mass in sphenoidal and cavernous sinuses accompanying sphenoidal bone erosion.

Case presentation.

A 71-year-old woman visited our hospital with left ophthalmalgia, ptosis, and diplopia. Neurological findings revealed left oculomotor, trochlear and abducent nerve palsies. Endocrine tests indicated partial hypopituitarism. Initial CT and MRI revealed that a mass in sphenoidal and cavernous sinuses had invaded the sella with osteolysis of the sphenoid bone. At around four weeks, almost all the symptoms of cranial nerve palsies were relieved. Seven weeks later, she had a high fever and cervical lymph node (CLN) swellings. CLN biopsy revealed CD20-positive B-cells. She was diagnosed with diffuse large B-cell lymphoma (DLBCL). ^18^F-fluorodeoxyglucose positron emission tomography/computed tomography (PET/CT) revealed elevated uptake at the erosion lesion of the sphenoidal bone, but not the pituitary gland. After chemotherapy, all the symptoms related to systemic lymphoma were relieved, but partial hypopituitarism remained. The mass in sphenoidal and cavernous sinuses and elevated uptake by PET/CT were dissolved.

**Conclusion:**

This case of DLBCL had a unique clinical course; initial presentation of hypopituitarism and symptoms mimicking THS. There was also rare demonstration of mass lesions related to DLBCL in the sphenoidal and cavernous sinuses compressing the pituitary gland through an eroded area of the sphenoidal bone. It should be clinically cautioned that DLBCL can be associated with erosion of the sphenoidal bone and cause both hypopituitarism and THS-mimicking symptoms.

## Background

Diagnosis of lymphoma is likely often delayed because it is challenging to differentiate the area of involvement and to estimate the timing when symptoms present. Tolosa-Hunt syndrome (THS) is a benign disease with unilateral orbital pain with paresis of one or more of the third, fourth, and/or sixth cranial nerves [1]. THS-mimicking symptoms are clinically often encountered, and could be associated with malignant disease. Therefore, the differentiation from other diseases is critical. Generally, if THS-mimicking symptoms persist or worsen, the infiltrative neoplastic diseases are implicated as a cause of the symptoms; however, if they improve, benign diseases are considered [2].

Hypopituitarism is usually associated with pituitary mass lesions, whereas upper unilateral cranial nerve palsies are associated with skull base lesions around the cavernous sinus [2]. These two lesions are anatomically separated by the bone. Therefore, to understand the patient’s clinical state, it is radiologically important to investigate how these two lesions could be simultaneously involved. As for lymphoma originating from extra pituitary gland, only one case of nasopharyngeal lesion has been reported; there was both oculomotor nerve palsy and pituitary dysfunction, but no bone erosion [3].

Here, we present a case of diffuse large B-cell lymphoma (DLBCL) with a unique clinical course. Initially there was hypopituitarism, and there was ophthalmoplegia and unilateral upper cranial nerve palsies mimicking THS with sphenoidal and cavernous sinuses lesion invading the sella with erosion of the sphenoidal bone. She later presented typical symptoms of systemic lymphoma after the remission of cranial nerve symptoms. This case emphasizes the importance of considering DLBCL as a cause of hypopituitarism if there is association with THS-like symptoms, even when symptoms are temporary and there is no obvious mass originating from the pituitary gland.

## Case presentation

**Fig. 1:**
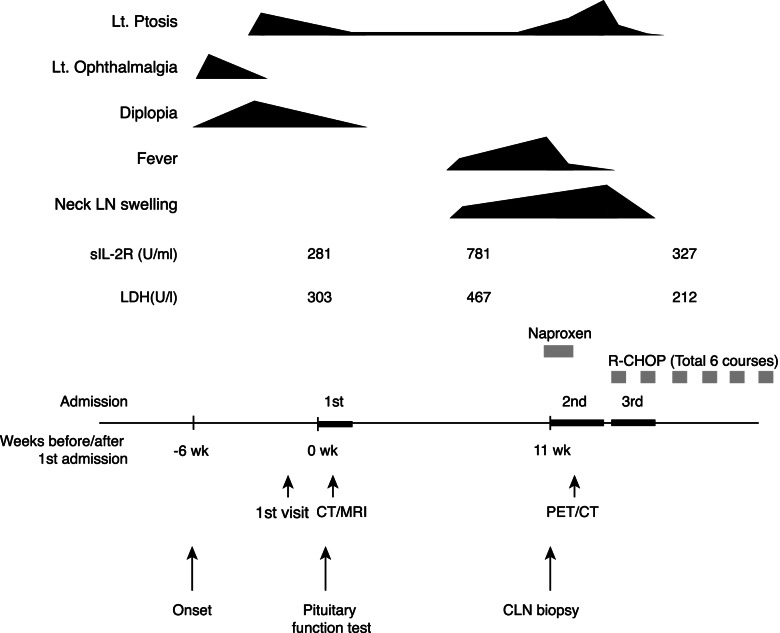
Clinical course

A 71-year-old woman visited our hospital with a complaint of left ophthalmalgia, left ptosis, and diplopia. Around six weeks before the first admission, she suddenly noticed left ophthalmalgia. A few days later, she noticed diplopia in all directions when she drove a car. Left ophthalmalgia had been gradually waned, but she noticed left ptosis (Fig. 1). She had a history of chronic sinusitis, and a nasal voice had been persisted for several years. Alteration in visual field was not presented. On the first visit, neurological findings revealed left oculomotor, trochlear and abducent nerve palsies. Her pupils were round, not anisocoric, and prompt consensual and direct light reflexes. Pupillary reflexes were bilaterally normal. Basal laboratory test suggested the central hypothyroidism (TSH 1.37 μIU/ml (reference range, 0.61–4.23 μIU/ml), Free T3 (FT3) 1.10 pg/ml (reference range, 2.52–4.06 pg/ml), Free T4 (FT4) 0.33 ng/dl (reference range, 0.75–1.45 ng/dl). Two weeks later, she was admitted to our hospital. In the physical examination, her body mass index was 21.2 kg/m^2^ and blood pressure was 121/62 mmHg. Neither swelling nor of lymph node pain was founded. Slight left ptosis and diplopia (horizontal) remained, but the symptoms were improved compared with those at the first visit. The nasal voices persisted, but no other definite abnormalities were detected in physiological or neurological examination.

**Fig. 2:**
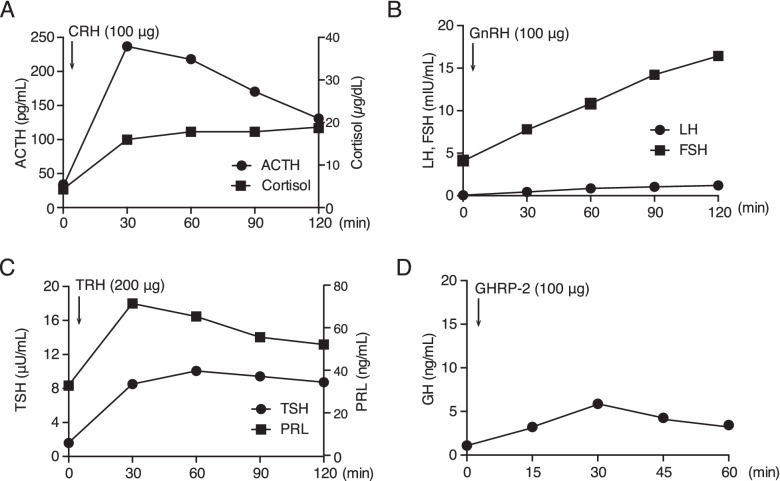
Pituitary function test at the first admission. **A**, **B**, **C**, **D** represent the results after CRH, GnRH, TRH, GHRP-2 load, respectively

Initial laboratory test results were within the normal range of ACE, sIL-2R and IgG4, suggesting no obvious evidence of infiltrative disease such as sarcoidosis, lymphoma or IgG4-related disease (Fig. 1 and Table 1). Biochemistry results were otherwise unremarkable, as shown in Table 1. Basal endocrine test showed a decrease in FT3, FT4, and LH (Table 1). Low levels of FT3 and FT4 with anti-Tg antibody positivity suggested the presence of Hashimoto's disease. However, sustained TSH level for low FT3 and FT4 levels suggested the co-presence of central hypothyroidism. To investigate the pituitary function further, CRH (100 μg) /TRH (200 μg) /GnRH (100 μg) and GHRP-2 (100 μg) load tests were performed (Fig. 2). ACTH and cortisol showed normal reactions after CRH load. TSH had a peak value of more than 6 μIU/ml, but a delayed response to the peak value after TRH load, suggesting central hypothyroidism. Both LH and FSH had a delayed response to the peak value after GnRH load, suggesting central hypogonadism. GHRP-2 load test revealed a low peak value of GH (less than 9 ng/ml), suggesting severe adult GH deficiency. Together, pituitary endocrine test revealed partial hypopituitarism. Oral administration of 25 μg levothyroxine per day was initiated.

**Table 1:** Laboratory Data on Admission

Urinary Data		Reference Range	Biochemical data/Immunology		Reference Range
pH	6.0	5.0–8.0	FPG	87 mg/dl	70–109
Prot	-	-	HbA1c	6.1%	4.6–6.2
Glu	-	-	TG	173 mg/dl	50–149
Osm	367 mOsm/L	< 850	HDL-C	31 mg/dl	40–96
Blood Count / Biochemical data			LDL-C	98 mg/dl	70–139
WBC	3610 /μl	3500–9100	CRP	0.17 mg/dl	≦0.14
neutrophil	1770 /μl		sOsm	286 mOsm/L	276–292
lymphocyte	1430 /μl		TPOAb	< 3 IU/ml	< 3.3
eosinophil	150 /μl		TgAb	71 U/ml	< 19.3
basophil	30 /μl		GH	1.6 ng/ml	
RBC	397 × 10^4^ /μl	376–500 × 10^4^	IGF-I	106 IU/ml	57–135
Hb	12.8 g/dl	13.5–17.6	PRL	34.3 ng/ml	
Ht	32.4%	33.4–44.9	LH	0.3 IU/ml	
Plt	23.5 × 10^4^ /μl	13.0–36.9	FSH	5.9 IU/l	
TP	6.1 g/dl	6.7–8.3	Estradiol	< 5 pg/ml	
Alb	3.9 g/dl	3.8–5.2	TSH	2.34 μIU/ml	0.61–4.23
GOT	26 U/l	10–40	Free T3	1.27 pg/ml	2.52–4.06
GPT	18 U/l	5–40	Free T4	0.44 ng/dl	0.75–1.45
LDH	299 U/l	124–222	ACTH	47.3 pg/ml	
CK	218 U/l	45–163	Cortisol	11.9 μg/dl	
UA	3.9 mg/dl	2.5–7.0	AVP	0.5 pg/ml	
BUN	9.1 mg/dl	8.0–22.0	IgG	1030 mg/dl	870–1700
Cr	0.70 mg/dl	0.47–0.79	IgG4	24 mg/dl	11–121
eGFR	62.3 ml/min/1.73m^2^	≧60	PR3-ANCA	< 0.5 IU/ml	< 3.5
Na	139 mEq/l	136–147	MPO-ANCA	< 0.5 IU/ml	< 3.5
K	4.0 mEq/l	3.6–5.0	ACE	11.9 U/L	8.3–21.4

**Fig. 3:**
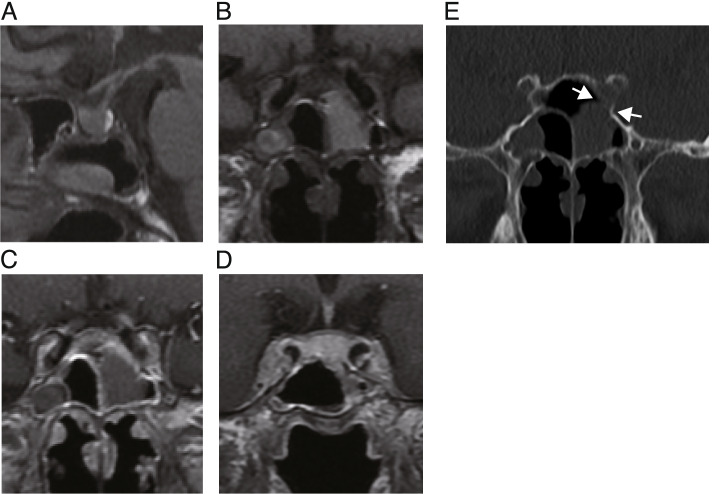
MRI **A**-**D** and CT **E** images at the first admission. **C**, **D** MRI performed after injection of gadolinium. **E** Space between arrows indicates the erosion of the sphenoidal bone

Initial magnetic resonance imaging (MRI) of the hypothalamus and pituitary gland indicated a heterogeneous enhancement of supra-sellar regions with a mass on the left sphenoid sinus wall and involvement of the left cavernous sinus (Fig. 3A-D). The pituitary gland was homogeneously enhanced, and slightly compressed by the infiltrated mass from the sphenoidal sinus. The stalk was median and not enlarged (Fig. 3A and D). Hyperintensity of posterior pituitary on T1 weighted intensity was preserved (Fig. 3A). Purulent reservoir and polyp-like mass were detected in the left sphenoidal and frontal sinuses. CT also showed the purulent reservoir in left sphenoidal and frontal sinuses, as shown in MRI. Furthermore, the osteolytic lesion of the sphenoid bone was observed as shown in Fig. 3E. These images suggested the presence of the infiltrative neoplastic lesion. However, because all the symptoms related to cranial nerve palsies were gradually waned, biopsy of the mass at the sphenoidal sinus was not performed based on the discussion with the patient with specialists of the otorhinolaryngology and neurosurgery. At around four weeks after the first admission, most of the symptoms had disappeared, except very slight left ptosis (Fig. 1).

**Fig. 4:**
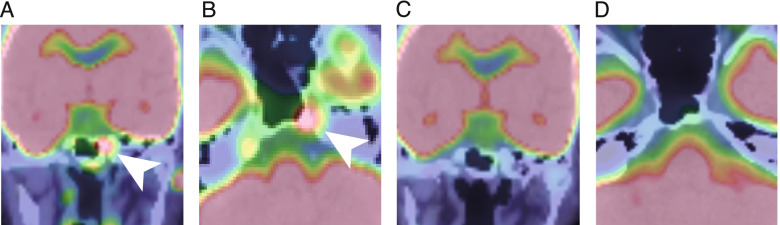
^18^F-fluorodeoxyglucose positron emission tomography images at the second admission **A** and **B** and after six courses of treatment with R-CHOP **C** and **D**. Arrowheads in A and B indicate elevated uptake at left sphenoidal bone lesion

Around seven weeks later, she noticed the worsening of the left ptosis and had high fever, cervical lymph node (CLN) swellings, and night sweats. She was secondary admitted to our hospital (Fig. 1). Laboratory tests revealed elevated serum sIL-2R (781 U/ml, reference range, 122–496 U/ml) and LDH (467 U/l, reference range, 124–222 U/l) levels. Since hematological or otorhinolaryngological disease was strongly suspected, CLN biopsy was performed. Immunohistological examination of CLN revealed the presence of CD20, BCL-6, focal CD5 positive but CD23 and cyclin D negative B-cells. Cell proliferation index Ki-67 was about 90%. Based on the pathophysiological findings, she was diagnosed as having DLBCL. In cerebrospinal fluid analyses, cytology examination revealed no evidence of malignant cells. ^18^F-fluorodeoxyglucose positron emission tomography/computed tomography (PET/CT) revealed elevated uptake in systemic lymph nodes, including the left sphenoidal (Fig. 4A and B) and cervical lesions, which corresponded with the osteolytic lesion of the sphenoid bone and CLN swelling lesions, respectively, but not in the pituitary gland. After diagnosis of DLBCL, she was treated with cyclophosphamide, doxorubicin hydrochloride, vincristine sulfate, and dexamethasone plus rituximab (R-CHOP) for six courses in total. After initiation of chemotherapy, there was improvement of all the symptoms associated with systemic lymphoma except hypopituitarism. Elevated serum sIL-2R and LDH levels were decreased to within the normal range (Fig. 1).

Ten months after the initiation of chemotherapy, endocrine function test was re-examined and revealed sustained severe GH deficiency and central hypogonadism, but improvement of central hypothyroidism. After cessation of levothyroxine, her thyroid function remained within normal range (TSH 3.19 μIU/ml (reference range, 0.61–4.23 μIU/ml), free T4 0.98 ng/dl (reference range, 0.75–1.45 ng/dl)). MRI showed intact pituitary and nasopharyngeal site. PET/CT showed almost complete remission of elevated uptake at the left sphenoidal lesion (Fig. 4C and D). One year after the treatment, there has been no recurrence of DLBCL nor cranial nerve palsies.

## Discussion and conclusions

This case of DLBCL showed both a unique clinical course with endocrinological impairment, and rare neurological images demonstrate the anatomical features to explain the clinical symptoms. Our diagnostic workup is here: Our patient initially presented the symptoms of both hemi-upper cranial nerve palsies mimicking THS and hypopituitarism with erosion of the sella and compressed pituitary gland by a mass from the sphenoidal sinus. THS-like symptoms spontaneously disappeared at around ten weeks after the onset. During the first admission, we differentially diagnosed the diseases that could cause THS-mimicking symptoms, unilateral ophthalmoplegia with upper cranial nerve palsies. Such diseases are wide-ranging and include aneurysms, tumors, orbital inflammation, or nonspecific granulomas [1, 4]. Among these diseases, osteolytic lesions and recovery of THS-like symptoms suggested either some rare benign diseases such as sphenoid mucocele, osteomyelitis, tuberculosis, and allergic fungal rhinosinusitis, or specific infiltrative tumors like malignant lymphoma. After remission of the THS-like symptoms, however, there was a presentation of common symptoms related to systemic lymphoma including high fever and swelling of cervical LN. At this point, we could perform the cervical LN biopsy and diagnosed DLBCL.

One feature of our case is the simultaneous presentation of both hemi-cranial nerve palsies and hypopituitarism with compression of the pituitary gland by a mass from sphenoidal sinus though osteolytic lesion of the sphenoidal bone. Zahedi et al. reported a case of B-cell lymphoma at nasopharyngeal site in which there was presentation of both pan-hypopituitarism and oculomotor nerve palsy [3]. They also reviewed 17 cases of nasopharyngeal B-cell lymphoma, but none of the cases of nasopharyngeal B-cell lymphoma presented cranial nerve palsies nor hypopituitarism [3]. Similarly, Wajima et al. reviewed 15 cases of sphenoidal lymphoma [5]; 73.3% patients initially presented with ptosis or diplopia caused by II, III, IV, or VI palsy, but not hypopituitarism. Furthermore, they reported that the most commonly extended area of tumor was the cavernous sinus (33.3%) as shown in our case [5]. Together, it is clinically important to consider that sphenoidal lymphoma could simultaneously cause both hypopituitarism and hemi-cranial nerve palsies.

Another feature of our case is the erosion of the sphenoidal bone. Etiologically, besides neoplastic disease, rare benign diseases such as sphenoid mucocele, osteomyelitis, tuberculosis, and allergic fungal rhinosinusitis are known to cause bone erosion at the paranasal site [6–9]. Although the sphenoid sinus is a rare location for mucocele, it could eventually cause hypopituitarism and cranial nerve palsies accompanied by bone erosion and destruction [8, 10]. Anatomically, since sphenoidal bone composes sella and cavernous sinus, it is a critical structure to protect pituitary gland and upper proximal cranial nerves running through the cavernous sinus [11, 12]. Only one report has shown a case with both hypopituitarism and cranial nerve palsy by lymphoma at the paranasal site without definite bone erosion [3]. Based on the anatomical features, it was difficult to explain both symptoms being caused by one lesion from the nasopharyngeal site [3]. On the other hand, in our case the mass was shown to originate from the sphenoidal or cavernous sinus and it directly compressed the pituitary gland and cranial nerve roots through bone erosion site to induce both symptoms. Although it is still hard to clearly judge if the pituitary gland was not originally involved, since PET/CT showed elevated uptake at the sphenoidal bone erosion site but not in the pituitary gland, the effect of the invaded mass could be emphasized.

Another feature of our case is the spontaneous recovery from THS-like symptoms at around ten weeks after the onset. Since the diagnostic criteria for THS comprises ophthalmalgia, persistent symptoms, and spontaneous remission, THS was one of the potential candidates of differential diagnosis at the first admission. One of the reasons why these symptoms were relieved might be owing to promotion of steroid secretion by CRH load, which was conducted to evaluate pituitary function, and this might react to relieve the symptoms of lymphoma, although some symptoms had already been spontaneously waned at this point.

A limitation of our case is that we could not directly show immunohistological evidence of the invaded mass in the sinuses. We planned to conduct pathological diagnosis by biopsy of the lesion and whole-body examination by PET/CT at the first admission. However, as the symptoms had been relieved, the mass in the sinus was considered to be more likely due to chronic sinusitis, and biopsy of the mass and PET/CT were not performed after the discussion with the patient and the relevant departments. Therefore, we judged that her symptoms due to mass at the paranasal site were related to DLBCL based on the immunohistological diagnosis of CLN biopsy, and on her clinical course with the rapid and well-marked response to R-CHOP therapy.

In conclusion, this is a case of DLBCL with a unique clinical course; initial presentation of both hypopituitarism and unilateral cranial nerve palsies, and ophthalmoplegia with erosion of the sphenoidal bone. After the remission of cranial nerve symptoms, common systemic symptoms of lymphoma appeared, eventually leading to diagnosis. It is clinically important to remember that bone erosion of sphenoidal lesion related to DLBCL could cause both hypopituitarism and THS-mimicking symptoms due to its anatomical features.

## Data Availability

The datasets used and analyzed during the current study are available from the corresponding author on reasonable request.

## Abbreviations

CLN: Cervical lymph node
DLBCL: Diffuse large B-cell lymphoma
MRI: Magnetic resonance imaging
PET/CT: ^18^F-fluorodeoxyglucose positron emission tomography/computed tomography
R-CHOP: Cyclophosphamide, doxorubicin hydrochloride, vincristine sulfate, and dexamethasone plus rituximab
THS: Tolosa-Hunt syndrome
